# Dystroglycan-HSPG interactions provide synaptic plasticity and specificity

**DOI:** 10.1093/glycob/cwae051

**Published:** 2024-09-02

**Authors:** James Melrose

**Affiliations:** Raymond Purves Bone and Joint Research Laboratory, Kolling Institute, St. Leonards, NSW 2065, Australia; School of Medical Sciences, Faculty of Medicine and Health, The University of Sydney at Royal North Shore Hospital, St. Leonards, NSW 2065, Australia; Graduate School of Biomedical Engineering, Faculty of Engineering, University of New South Wales, Sydney, NSW 2052, Australia

**Keywords:** cell-extracellular matrix communication, dystroglycan, heparan sulphate proteoglycans, neurotransmission, phototransmission

## Abstract

Aim: This study examined the roles of the laminin and proteoglycan receptor dystroglycan (DG) in extracellular matrix stabilization and cellular mechanosensory processes conveyed through communication between the extracellular matrix (ECM) and cytoskeleton facilitated by DG. Specific functional attributes of HS-proteoglycans (HSPGs) are conveyed through interactions with DG and provide synaptic specificity through diverse interactions with an extensive range of cell attachment and adaptor proteins which convey synaptic plasticity. HSPG-DG interactions are important in phototransduction and neurotransduction and facilitate retinal bipolar-photoreceptor neuronal signaling in vision. Besides synaptic stabilization, HSPG-DG interactions also stabilize basement membranes and the ECM and have specific roles in the assembly and function of the neuromuscular junction. This provides neuromuscular control of muscle systems that control conscious body movement as well as essential autonomic control of diaphragm, intercostal and abdominal muscles and muscle systems in the face, mouth and pharynx which assist in breathing processes. DG is thus a multifunctional cell regulatory glycoprotein receptor and regulates a diverse range of biological and physiological processes throughout the human body. The unique glycosylation of the αDG domain is responsible for its diverse interactions with ECM components in cell-ECM signaling. Cytoskeletal cell regulatory switches assembled by the βDG domain in its role as a nuclear scaffolding protein respond to such ECM cues to regulate cellular behavior and tissue homeostasis thus DG has fascinating and diverse roles in health and disease.

## Introduction

Dystroglycan (DG) is a multifunctional cell adhesion laminin receptor composed of covalently linked α extracellular and cytoplasmic β domains that act not only as an anchorage for cells to the ECM but it also modulates outside-in cell signaling (Bello and Darribère 2016). αDG is a ubiquitous neural receptor in skeletal muscle, CNS/PNS, digestive tract, kidney, skin and reproductive system binding to laminins, agrin and perlecan in muscle and brain ECM and with slit proteins in the spinal cord, neurexin in synapses and pikachurin in the retina (Marrone et al. 2011; Nickolls and Bönnemann 2018; Lindenmaier et al. 2019; Jahncke and Wright 2023; Sciandra et al. 2023). Interaction of αDG with HSPGs conveys some of its key functional attributes (Gee et al. 1994; Jacobson et al. 2001; Jahncke and Wright 2023). αDG is a heavily glycosylated protein with glycan representing over 50% of its mass however brain αDG has reduced glycosylation and ligand binding affinity compared to muscle αDG reflecting differing tissue-specific functional duties. αDG interaction with agrin and perlecan supports the localization of acetylcholinesterase and acetylcholinesterase receptors in motor neurons which regulate conscious and autocrine neuromuscular activity (Gee et al. 1994; Jacobson et al. 2001; Saito et al. 2003). The α-domain of DG has unique glycosylation patterns that facilitate interaction with ECM components (Briggs et al. 2016). DG has a unique sugar composition including the presence of two ribitol phosphates on the matriglycan component of DG (Manya et al. 2016). The muscular dystrophy gene to a trans-membrane protein (TMEM) encodes a ribitol-β1,4-xylosyl transferase biosynthetic enzyme responsible for attachment of the Xylβ1-4Rbo5P disaccharide to the O-mannosyl linkage region and is also responsible for the elongation of the 3GlcAβ1-3Xylα1- units on the mannosyl chain (Praissman et al. 2016; Okuma et al. 2023). Thus TMEM5 acts as a UDP-D-xylose:ribitol-5-phosphate β1,4-xylosyltransferase. This provides an essential aspect of the functional glycosylation of DG in health and disease (Goddeeris et al. 2013). The glucuronyl-transferase B4GAT1 is required for initiation of LARGE-mediated αDG functional glycosylation (Praissman et al. 2014; Willer et al. 2014).

αDG interacts with agrin, neurexin, perlecan, eyes-shut and pikachurin (Ibraghimov-Beskrovnaya et al. 1992; Gee et al. 1994; Sugita et al. 2001; Sato et al. 2008) in neural and ocular tissues conveying synaptic stabilization, synaptic plasticity and specificity of action. The cytoplasmic β-domain of DG interacts with adaptor and cytoskeletal proteins such as ezrin that regulate cytoskeletal organization acting as molecular switches for the transmission of ECM to the cell (Sciandra et al. 2023) regulating cell signaling through the cytoskeletal protein dystrophin (Ibraghimov-Beskrovnaya et al. 1992; Suzuki et al. 1994; Jung et al. 1995).

## Dystroglycan and mechanotransduction

**Fig. 1:**
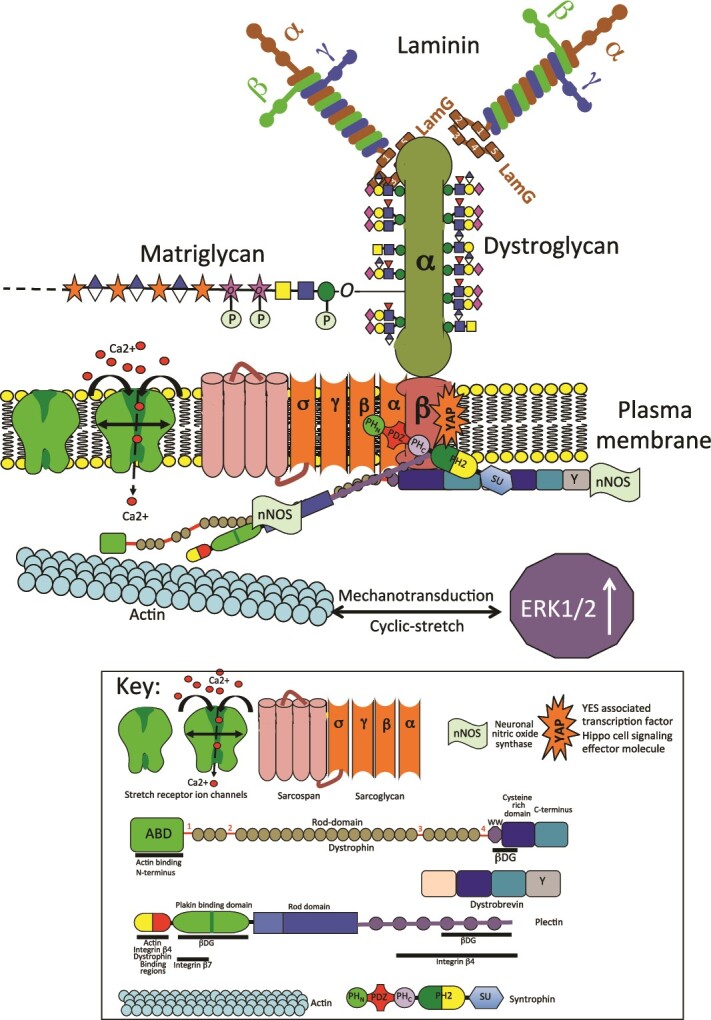
Schematic depiction of the structural organization of the dystroglycan laminin receptor showing it’s covalently linked α and β domains, extensive glycosylation of the α-domain, and sarcospan, sarcoglycan glycoproteins and stretch receptors that interact with the αDG domain. βDG interactive cytoplasmic proteins including dystrophin, plectin, dystrobrevin, syntrophin form an organizational instructive scaffold of importance in cell signaling. This scaffold interfaces with the actin cytoskeleton which transfers cyclic stretching and relaxation that regulates extracellular signal-regulated kinase 1/2 (ERK1/2) cell signaling. An influx of Ca2+ through stretch activated ion-channels regulate neuronal nitric oxide synthase (nNOS) activity, a signaling molecule that provides synaptic plasticity. This also regulates the cerebrovasculature of the neurovascular unit in the CNS/PNS and regulates brain perfusion. Plectin has actin, dystrophin, integrin and βDG binding sites and forms an interactive instructional scaffold with important roles in cell-signaling. βDG also has a binding site for yes associated protein (YAP), a transcription factor effector of the hippo cell signaling pathway. This is a mechanosensitive cell signaling pathway that regulates tissue composition, aids in the homeostasis of tissues and also regulates the final size attained by organs in maturity.

**The dystrophin-glycoprotein complex has a central role to play** in mechanotransduction (Campbell and Kahl 1989). DG complexes with dystrophin, sarcoglycan-sarcospan and syntrophin to facilitate mechanotransductive processes (Fig. 1). Mechano-transducer accessory proteins such as neuronal nitric oxide synthase (Garbincius and Michele 2015) and YAP also attach to dystrophin, a PPxY binding motif sequesters YAP (McNally et al. 1998; Garbincius and Michele 2015; Morikawa et al. 2017). Dystrophin also regulates mechanosensitive ion channels including stretch-activated Ca^2+^ channels and *tr*ansient receptor potential cation *(*TRPC) channels (Millay et al. 2009). Cyclic stretch activates ERK1/2 and 5′ cyclic monophosphate (AMP)-activated protein kinase (AMPK) signaling pathways via the DG glycoprotein complex and an associated protein, plectin which forms a mechanotransductive scaffold in conjunction with DG (Takawira et al. 2011; Winter and Wiche 2013).

## βDG interactome

**Fig. 2:**
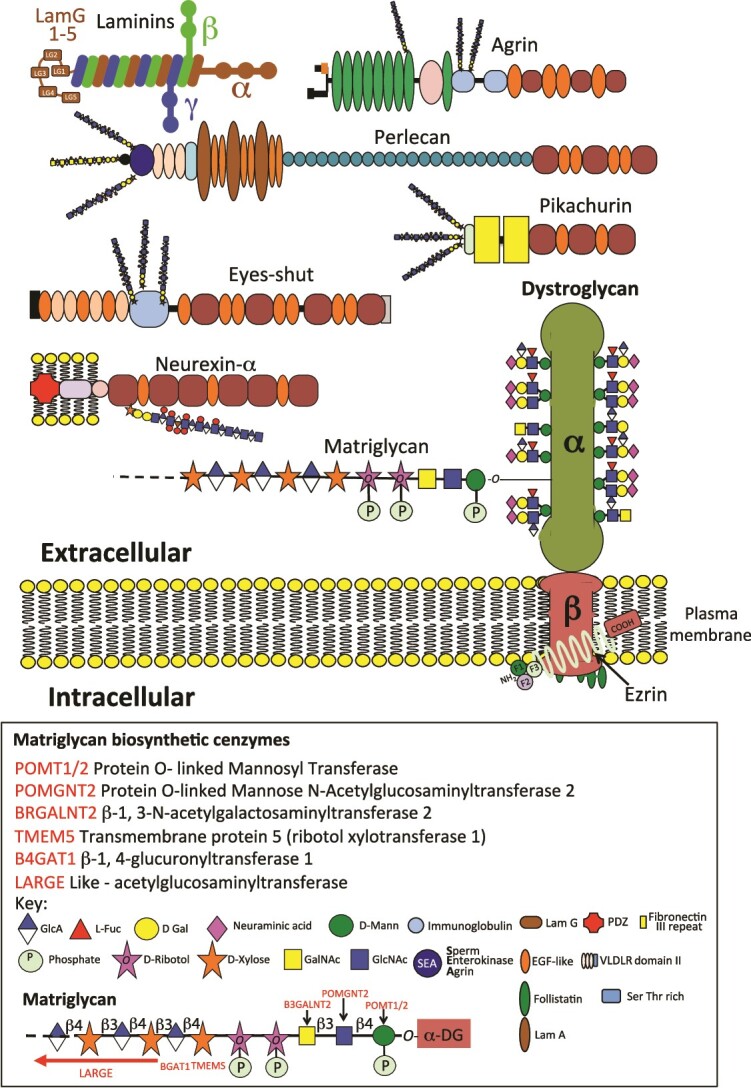
Schematic depiction of the DG laminin receptor and some of its interactive ligands. Some of the extracellular HSPG ligands that interact with αDG are shown including, perlecan, agrin, pikachurin and eyes-shut. Biosynthetic enzymes responsible for the glycosylation of the α-dystroglycan matriglycan component are also shown emphasizing the unique glycosylation of DG which conveys its unique interactive properties with ECM components.

βDG has roles as a nuclear scaffolding protein (Sciandra et al. 2024) and is localized with MEK (mitogen activated protein kinase) in membrane ruffles and with ERK (Extracellular signal-regulated kinase) in focal adhesions in fibroblasts. MEK is a serine/tyrosine/threonine kinase that phosphorylates and activates MAPK (mitogen-activated protein kinase) while ERK is a serine/threonine-specific protein kinase, ERK signaling represents the culmination of the MAPK cascade. ERK/MAPK signaling is essential in the development of the nervous system from neuroprogenitor cell populations (Iroegbu et al. 2021). Interaction of βDG with ezrin regulates cytoskeletal organization (Iroegbu et al. 2021) (Fig. 2). The long arm of laminin contains five homologous A chain LamG domains that interact with integrins α6β1 (Aumailley et al. 1990), α7β1 (Kramer et al. 1991), and α3β1 (Gehlsen et al. 1992) and HS in ECM and cell surface HSPGs (Yurchenco et al. 1990). The dystrophin scaffold interacts with stretch receptors regulating ion channels and the influx of Ca2+ into the neuron. Ca 2+ is a universal second messenger in neurons regulating membrane depolarization and neuronal activation. Neuronal nitric oxide synthase (nNOS) also binds to the dystrophin-actin cytoskeletal scaffold and regulates the cerebral vasculature (Melikian et al. 2009; Förstermann and Sessa 2012; Costa et al. 2016) and regulates the perfusion of the brain (O'Gallagher et al. 2022). Cyclic stretching conveyed through the actin cytoskeleton regulates ERK1/2 signaling (Wortzel and Seger 2011).

## Glycosyl sulfotransferases modulate bioresponsive HSPG regulatory properties

Spatio-temporal glycosylation patterns of ECM components drive developmental processes and maintain tissue homeostasis (Basu et al. 2022) in health and disease (Reily et al. 2019) emphasizing the significant roles of glycosyltransferases in the determination of tissue form and function (Mehboob and Lang 2021). The link between the unique glycosylation patterns of DG and muscular dystrophies is a good example of how aberrant glycosylation of key ECM effector molecules can effect normal tissue function. HS synthesis in tissues is complex and gives rise to side chains in HSPGs that vary in sequence and sulfation patterns providing an extraordinary diversity in the ligand interactive capability of HSPGs in the glycocalyx and ECM (Cummings 2009). Spatio-temporally expressed glycosylation patterns (Basu et al. 2022) drive tissue morphogenesis and also maintain tissue homeostasis and function (Reily et al. 2019) emphasizing the modulatory roles of glycosyltransferases in normal and pathological tissues (Nogami et al. 2004). Subtle control of HS structure can modulate biological responses in-situ (Bishop et al. 2007; Basu et al. 2022) through interactions with structural ECM proteins, cell adhesive glycoproteins, integrins, receptors (Gopal et al. 2021) and chemokines (Gordts and Esko 2015).

## Dystroglycan-HSPG interactions of importance in vision

DG has roles in the development and function of the nervous system (Jahncke and Wright 2023) and affects the structural remodeling of dendritic spines (Figiel et al. 2022). Pikachurin, synthesised by photoreceptors, forms a key post-synaptic DG signaling complex downstream of ON-bipolar neurons (Orlandi et al. 2018) and regulates functional synaptic connection between retinal photoreceptor and bipolar cells (Omori et al. 2012). Pikachurin also has roles in the formation and stabilization of the photoreceptor ribbon synapse through interaction with DG. Furthermore, LRRTM synaptic adhesion molecules also have roles in retinal synapse formation, signaling specificity and stabilization through interactions with HSPGs. LRRTM4 is enriched in retinal rod bipolar cells which connect with rod photoreceptors to facilitate neurotransmission and phototransductive processes in vision (Agosto and Wensel 2021). To date, only one highly conserved DG gene has been discovered in mammals (Henry and Campbell 1999). DG has many roles in CNS/PNS neural circuit development (Jahncke and Wright 2023) α is a peripheral membrane lectin-like glycoprotein, with an abundance of *O*-linked glycan in its αDG extracellular domain while the membrane-spanning β-DG domain organizes cytoskeletal proteins with roles in cell signaling (Ervasti and Campbell 1991). The mucin-rich region of α-DG is rich in serine and threonine residues substituted with ~40 *O*-Man and *O*-GalNAc residues (Gomez Toledo et al. 2012; Harrison et al. 2012) and matriglycan (Goddeeris et al. 2013) which may exceed 100 disaccharides in length. These residues interact with LamG domains in HSPGs (Hohenester 2019) linking αDG to the basement membrane (Yoshida-Moriguchi and Campbell 2015) and also aid in the stabilization of the photoreceptor ribbon synapse and ciliary axenome through interactions mediated by eyes-shut and pikachurin.

## Eyes shut and Pikachurin

**Fig. 3:**
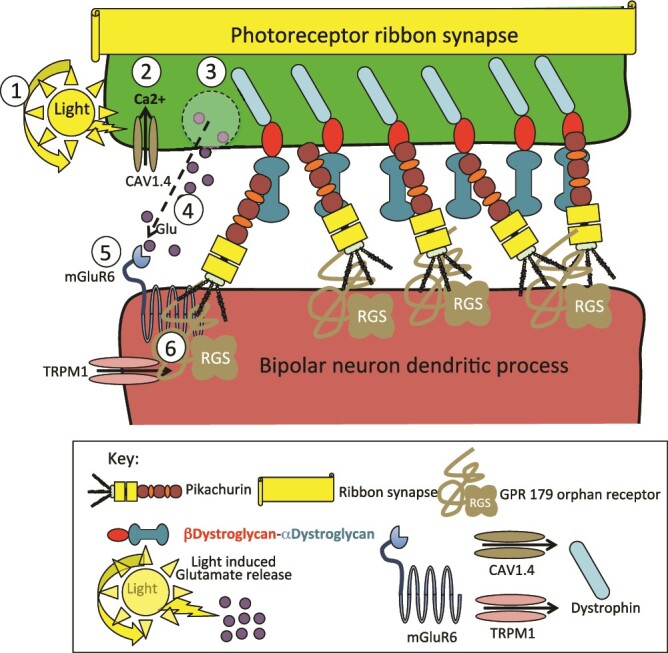
Schematic depiction of pikachurin interaction with dystroglycan and dystrophin in the photoreceptor ribbon synapse, GPR 158/179 orphan receptor and mGluR6 in neurotransduction with retinal bipolar neurons in visual processing. 1. Photons are captured as part of the phototransductive process which 2. Activates the voltage gated CAV1.4 Ca2+ channel. 3. The influx of Ca2+ activates transport processes of Glu neurotransmitter within synaptic vesicles which merge with the membrane of the synaptic gap. 4. This results in the release of glutamate into the synaptic gap. 5. Glutamate is taken up by the mGluR6 metabromic glutamate receptor on bipolar neurons in the retina. 6. Transient receptor potential cation channel subfamily M member 1 (TRPM1) regulates neurotransduction in activated bipolar neurons as part of visual processing. ON-bipolar cell dendrites express a unique metabotropic glutamate receptor 6 (mGluR6). TRPM1 is a mGluR6-coupled cation channel in retinal ON-bipolar cells that regulates G protein receptor (GPCR) coupled signaling. TRPMI1 is the end-point of the mGluR6 signal transduction cascade in bipolar neurons in the retina.

Human Eyes shut (EYS), contains five LamG domains, three of these are predicted to be functional Ca^2+^ binding sites (Yu et al. 2016; Lu et al. 2017). Mutations in *EYS* result in retinitis pigmentosa demonstrating the important roles it plays in vision (Abd el-Aziz et al. 2008, Collin et al. 2008). Over 100 unique missense variants have been detected, two of these affect a putative Ca^2+^ ligand binding site in LamG4. The interaction of pikachurin with α-DG provides synaptic stabilization between retinal photoreceptor and bipolar neurons essential for the processing of phototransductive and neurotransductive ocular signals and ocular vision (Sato et al. 2008). Pikachurin interacts with dystrophin-DG complexes, and GPR179 orphan receptor in bipolar neurons to facilitate phototransduction and neurotransduction of ocular signals to the brain (Fig. 3). GPR179 is specifically expressed in the retina, it forms complexes in native retinas with mGluR6 (metabotropic glutamate receptor 6) and TRPM1 (transient receptor potential cation channel subfamily M member 1), the main components of the signaling in ON-bipolar cells (ON-BC) to regulate G protein-coupled receptor (GPCR) signaling. ON-BCs are involved in spatial processing of the visual inputs from photoreceptors (Koike et al. 2010; Orlandi et al. 2012). Photoreceptor activation in response to light exposure results in an influx of Ca2+ through a CAV1.4 voltage gated calcium channel. This initiates synaptic vesicle mobilization and migration to the synaptic gap where membrane fusion of the synaptic vesicles releases glutamate neurotransmitter into the synaptic cleft between photoreceptors and ON bipolar cells. Glutamate is taken up by mGluR6 on the ON bipolar neuron initiating a G-protein coupled signaling cascade, mGluR6 and TRPM1 regulate this neurotransductive response (Kolb et al. 1995; Martemyanov and Sampath 2017), TRPM1 is enriched in ON bipolar neurons, GPR179 orphan receptor, is specifically expressed in retinal neurons and forms a complex with mGluR6 and TRPM1 regulating these interactions (Nomura et al. 1994; Koike et al. 2010; Morgans et al. 2010) and is also a ligand for pikachurin which in turn interacts with αDG to stabilise the ribbon synapse. The LG2-EGF-EGF-LG3 domains of pikachurin are responsible for its α-DG binding activity (Kanagawa et al. 2010). This interaction stabilizes the axonome primary cilium which attaches outer regions of photoreceptors and their inner regions.

## Neurexins provide synaptic stability, plasticity and specificity of interaction in neurotransduction and network signaling

**Fig. 4:**
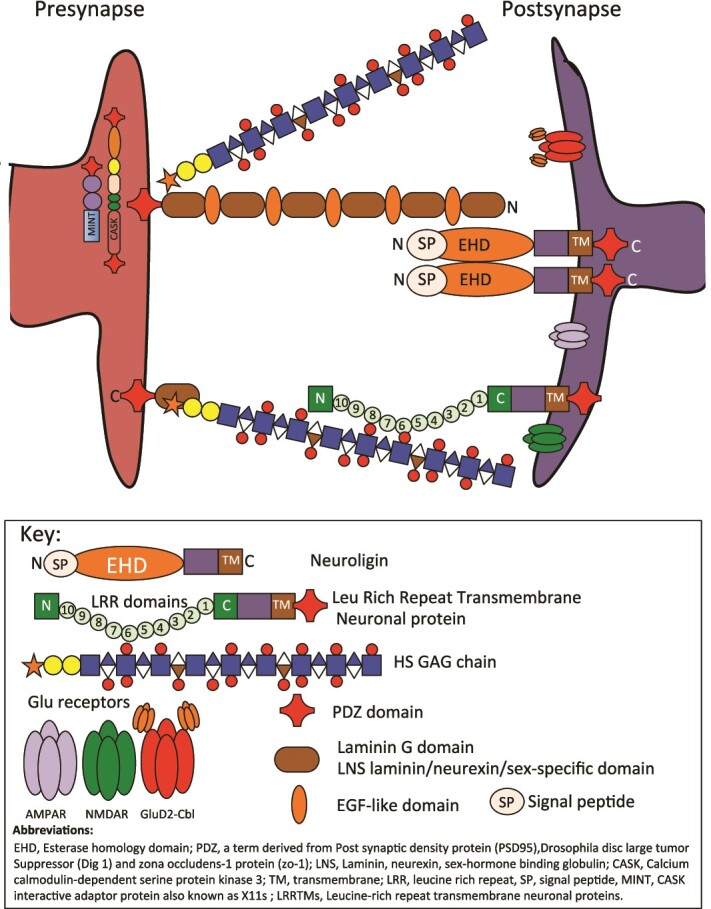
Schematic depiction of the synapse and proteins that interact with neurexins with roles in synaptic stabilization and which convey diverse interactions that provide synaptic specificity and plasticity in neurotransmission.

Neurexins are key organizers of synapses that perform specific functions that are essential for normal brain function (Reissner et al. 2013) and are presynaptic cell-adhesion receptors that occur as two principal forms, a longer α-neurexin and a shorter β-neurexin. α-Neurexin core proteins contain six Laminin G LNS (laminin/neurexin/sex-specific globulin) domains, these are interspersed with three EGF-like domains, an O-linked carbohydrate attachment region, and a cysteine-loop domain (Liu et al. 2018). α-neurexins, also contain a transmembrane region and a short cytoplasmic tail. β-Neurexins contain a short N-terminal β-neurexin-specific sequence spliced on to an α-neurexin LamG domain. Vertebrate neurexins undergo extensive alternative splicing producing thousands of isoforms (Ullrich et al. 1995; Treutlein et al. 2014), interactive with a vast range of binding partners through vast epitope coverage (Chowdhury et al. 2021). Examples of binding partners for the neurexins include the calcium/calmodulin-dependent serine protein kinase (CASK) (Stevenson et al. 2000; Hsueh 2006; LaConte et al. 2016; Pan et al. 2021; Dybus et al. 2023), the leukocyte common antigen-related receptor protein tyrosine phosphatases (LAR-RPTPs) (Lee et al. 2020), the neuroligins (Lisé and El-Husseini 2006; Bourne and Marchot 2014; Qin et al. 2020) and leucine-rich-repeat transmembrane neuronal proteins (LRRTMs) (Roppongi et al. 2017) and MINT proteins (Chatr-aryamontri et al. 2007; Ceol et al. 2010). MINTS regulate APP trafficking and βA generation, deletion of MINTS decreases βA plaque formation in AD models (Ho et al. 2008). MiNT 3 (Mitochondrial inner NEET protein) is an inner mitochondrial Fe-S protein with multiple roles in the regulation of Fe metabolism, free radical and ATP production in health and disease and due to the labile nature of the co-ordination of its two functional Fe-S clusters is a potential therapeutic target (Tamir et al. 2015; Lipper et al. 2018; Mittler et al. 2019; Molino et al. 2020). Many of these interactions with synaptic proteins are mediated by the glycosaminoglycan (GAG) side chains of the neurexins (Yamaguchi 2002; Zhang et al. 2018) and provide synaptic specificity (de Wit and Ghosh 2016; Condomitti and de Wit 2018; Zhang et al. 2018; Melo-Filho et al. 2024) (Fig. 4). Mutations in neurexin genes, in particular *NRXN1*, are associated with diverse neuropsychiatric disorders (Cuttler et al. 2021). Neurexin KO causes diverse synaptic phenotypes in a synapse-specific manner ranging from effects on synapse numbers to regulation of synaptic Ca^2+^-signaling and neuronal signal transduction (Chen et al. 2024). AMPAR, (α-amino-3-hydroxy-5-methyl-4-isoxazolepropionic acid glutamate receptors), NMDAR, (N-methyl-D-aspartate glutamate receptor) and the GluD2, (Glutamate receptor), ionotropic, delta 2 (Matsuda et al. 2010) contribute to the specificity of synaptic interactions, synaptic plasticity and neurotransmission (Chater and Goda 2014; Royo et al. 2022).

## Perlecan and Agrin

Binding of perlecan and agrin to α-DG involves multiple LamG domains (Fallon and Hall 1994), (Talts et al. 1999). Perlecan clusters with DG and AChRs on the surface of muscle cells in the NMJ, this interaction is Ca2+ dependant (Andac et al. 1999; Talts et al. 1999). Agrin and perlecan have important roles in the clustering of acetyl cholinesterase receptors and acetyl cholinesterase enzyme in NMJ neuron basal structures interacting with a wide range of structural and cell adhesive glycoproteins such as MusK and CollQ in NMJ assembly and function; Aldunate et al. 2004; Smirnov et al. 2005; Sigoillot et al. 2010; Hubbard and Gnanasambandan 2013; Li et al. 2018; Guarino et al. 2020; Uyen Dao et al. 2023). Perlecan, agrin, type XVIII collagen form stabilizing networks in basement membranes. HSPGs have important instructive roles in neural network development (Melrose et al. 2021) and in neurodegenerative processes (Pintér and Alpár 2022). HSPGs promote deposition in brain tissues of insoluble pathological protein aggregates that contribute to the pathogenesis of diseases of cognitive decline such as AD and PD (Melrose, J., Smith, MM.).

## Degradation of the neural ECM by MMPs in neurodegenerative conditions

ECM assembly is altered in schizophrenia and diseases of cognitive decline such as AD and PD (Sethi and Zaia 2017) due to defective assembly processes and excessive catabolism of ECM components by MMPs and ADAMTS metalloproteases. MMPs also have roles in the turnover of ECM components during ECM remodeling in tissue morphogenesis and normal tissue development (Brkic et al. 2015; Rempe et al. 2016) as well as in ECM repair processes. Post translational modification of proteoglycans by MMPs is a normal event in tissue growth and development (Mead et al. 2022). MMP activity is normally controlled by endogenous MMP inhibitory proteins (Gardner and Ghorpade 2003; Lukaszewicz-Zając et al. 2014; Caban et al. 2022) however when this system is dysregulated this can lead to neurodegenerative and psychiatric disorders through defective cellular activity and tissue function (Kim and Joh 2012; Sethi and Zaia 2017; Rivera et al. 2019). An intricate stabilizing network of CNS/PNS proteoglycans functionalize neuronal and astrocyte niche micro-environments in the brain optimizing cellular activity by preserving membrane polarization dynamics, ionic micro-environments, ion fluxes, neuronal activation and network neurotransduction (Melrose 2024). The neuron is an ion sensitive cell type and control of its ionic environment is required to optimise activity in neurotransmission and co-ordination of neural network activity.

Table 1 summarises the functional attributes of some HSPG-DG interactions.

**Table 1:** Multifunctional HSPGs interactive with dystroglycan.

Protein	Sensory processes affected	Function
Perlecan	Perlecan is cytoprotective and facilitates cell-ECM osmo-mechanosensory instructive cues properties.	The three LamG domains in domain V of perlecan interact with α-DG. Perlecan regulates diverse cellular processes(Whitelock et al. 2008), stabilises tissues, sequesters growth factors/morphogens, regulates tissue development and morphogenesis, cell proliferation/differentiation in chondrogenesis,vasculogenesis, osteogenesis, inflammation, cardiac development, and angiogenesis (Lord et al. 2014; Vincent et al. 2022). Perlecan has mechanosensory, osmoregulatory roles in weight bearing and tensional connective tissues (Zhao et al. 2020; Guilak et al. 2021) and acts as a shear flow biosensor for endothelial cells and osteocytes, regulates SMCs, vascular tone, blood pressure, bone assembly and homeostasis (Thompson et al. 2011; Wang et al. 2014; Wijeratne et al. 2016). Perlecan is a key component of the vascular ECM and maintains endothelial cell barrier function, it inhibits SMC proliferation and maintains vascular homeostasis. Perlecan has roles in the repair of diseased connective tissues (Arikawa-Hirasawa 2022; Hayes et al. 2022; Hayes and Melrose 2023; Zhao et al. 2023). More than 30 HSPG2 mutations lead to Schwartz-Jampel Syndrome (Lin et al. 2021).
Collagen XVIII	Assembly and stabilization of basement membranes	Collagen XVIII stabilises NMJ assembly and function, eye development and maintenance of the BBB basement membranes. Collagen XVIII levels are elevated in cerebrospinal fluid following traumatic brain injury (Chen et al. 2013) and associated with brain lesions(Mueller et al. 2007).
Agrin	Assembly and function of NMJ, regulation of cardiomyocyte function, bioresponsive mechanoreceptor regulated by Hippo cell signaling	Agrin initiates MuSK kinase activity, a receptor tyrosine kinase *and* a key regulator of NMJ development. Agrin interacts with LRPR, rapsyn and DOK-7 cytoplasmic adaptor protein (Burgess et al. 1999). The NMJ agrin-Lrp4-MuSK cell signaling pathway (Herbst 2020) is disrupted in congenital myasthenia syndromes, Schwartz-Jampel syndrome, Fukuyama-type congenital muscular dystrophy, amyotrophic lateral sclerosis, and sarcopenia. Impaired MuSK signaling causes severe muscle weakness in congenital myasthenic syndromes. DOK7 promotes NMJ regeneration after nerve injury (Kosco et al. 2023), neuronal agrin promotes myoblast proliferation(Gros et al. 2022). Neuronal LRP4 regulates synapse formation and synaptic plasticity (Karakatsani et al. 2017). Mechanosensitive Yap/Taz effectors of Hippo cell signaling regulate cardiomyocyte replication/regeneration through Agrin.
α, β, γ Neurexin	Pre/Post Synaptic organization and function	Neurexins have cell adhesive functional interactions with several hundred synaptic proteins providing diversity and specificity in synaptic activity (Dai et al. 2022; Traunmüller et al. 2023). Stabilization of neural pre-and post synaptic terminal interconnections promote neurotransduction efficiency and synaptic plasticity in neural networks (Kim et al. 2022). Neurexin multi-protein complexes regulate pre-synaptic voltage gated Ca channels and functional neuronal receptors (Noborn and Sterky 2023) in health and disease (Cuttler et al. 2021).
Eyes Shut (Eys)	Photoreceptor stabilization and function	Eyes shut interacts with matriglycan O-mannosyl glycans on α-DG to stabilise the photoreceptor ribbon synapse in phototransductive interactions with retinal bipolar neurons (Husain et al. 2006; Liu et al. 2020), maintain photoreceptor morphology and visual acuity. Mutations in *Eys* result in vision impairment in retinitis pigmentosa (Suvannaboon et al. 2022).
Pikachurin	Photoreceptor stabilization and function	Pikachurin stabilises the photoreceptor axoneme cilium and ribbon synapse (Sugita et al. 2015; Furukawa et al. 2020), interacts with orphan receptor GPR179 (Orlandi et al. 2012; Orlandi et al. 2018) and α-DG in phototransduction (Furukawa et al. 2020) involving bipolar neurons of the retinal neural network essential in vision (Sato et al. 2008; Omori et al. 2012; Orlandi et al. 2018; Furukawa et al. 2020). LamG-domains interact with α-DG.

## Conclusions

Dystroglycan is a fascinating multifunctional laminin receptor with important roles in cell-ECM signaling and the regulation of neuronal activity and control of cellular behavior in health and disease. While the major focus of the present review was to explore the role of DG in neural tissues it should be noted that DG also has equally important regulatory roles in many other human tissues. The role of DG in the assembly and function of the NMJ in neuromuscular regulation was also briefly commented on. The importance of DG in this area becomes apparent when DG activity is affected by mutation or trauma and exemplified by a number of muscular dystrophy disorders. Dysfunctional DG activity in so-called dystroglycanopathies are clear examples of DG’s importance in neuromuscular control and when deregulated its impact on conscious human movement. DG also has roles in autonomic co-ordinated control of the diaphragm, intercostal and abdominal muscles as well as muscle systems in the face, mouth and pharynx which all assist in breathing processes. This is but one example of the diverse roles of DG in other human tissues. A greater understanding of the diverse properties of DG is thus clearly relevant to many areas of human physiology and cellular regulation in health and disease.
